# The Amount of Nitrogen Used for Photosynthesis Modulates Molecular Evolution in Plants

**DOI:** 10.1093/molbev/msy043

**Published:** 2018-04-19

**Authors:** Steven Kelly

**Affiliations:** Department of Plant Sciences, University of Oxford, Oxford, United Kingdom

**Keywords:** photosynthesis, efficiency, selection, evolution

## Abstract

Genome and transcript sequences are composed of long strings of nucleotide monomers (A, C, G, and T/U) that require different quantities of nitrogen atoms for biosynthesis. Here, it is shown that the strength of selection acting on transcript nitrogen content is influenced by the amount of nitrogen plants require to conduct photosynthesis. Specifically, plants that require more nitrogen to conduct photosynthesis experience stronger selection on transcript sequences to use synonymous codons that cost less nitrogen to biosynthesize. It is further shown that the strength of selection acting on transcript nitrogen cost constrains molecular sequence evolution such that genes experiencing stronger selection evolve at a slower rate. Together these findings reveal that the plant molecular clock is set by photosynthetic efficiency, and provide a mechanistic explanation for changes in plant speciation rates that occur concomitant with improvements in photosynthetic efficiency and changes in the environment such as light, temperature, and atmospheric CO_2_ concentration.

## Introduction

Cells are built from macromolecules (proteins, RNA, DNA, phospholipids, and polysaccharides) that in turn are constructed from monomers (amino acids, nucleotides, fatty acids, and sugars). The majority of plants can biosynthesize all of the monomers and macromolecules they require from inorganic carbon (CO_2_) and nitrogen (NO_3_^−^/${\text{NH}}_{4}^{\text{+}}$) obtained from their environment. Of these two resources, nitrogen is scarcer and hence plant growth rate is generally nitrogen limited in both natural and agricultural environments (Ingestad and Lund 1979; Evans 1983; Ingestad and Ågren 1992; LeBauer and Treseder 2008). This limitation in growth is caused by the fact that synthesis of proteins required for photosynthetic carbon assimilation needs a substantial nitrogen investment (Evans 1989; Hohmann-Marriott and Blankenship 2011).

Photosynthetic nitrogen use efficiency (PNUE) is the amount of carbon that can be fixed per unit of nitrogen invested by the plant. Multiple disparate anatomical, physiological, and molecular factors contribute to variation in PNUE such that there is a large variation between different plant species (Rotundo and Cipriotti 2017). For example, plants that use the C_4_ photosynthetic pathway exhibit higher nitrogen use efficiency when compared with plants that use C_3_ photosynthesis. The cohort of changes that facilitated C_4_ evolution enabled plants to reduce resource allocation to photosynthetic machinery without causing a corresponding reduction in photosynthetic rate (Oaks 1994). Thus, C_4_ plants can achieve ∼50% higher rates of photosynthesis than C_3_ plants given the same amount of nitrogen (Evans and von Caemmerer 2000).

Nucleotide monomers (A, C, G, and T/U) differ in their biosynthesis requirements, with different nucleotides requiring different quantities of nitrogen atoms for their construction. Adenine and guanine require 5 nitrogen atoms for their biosynthesis, cytosine requires 3, and thymine/uracil only require 2. Although the sequence and abundance of proteins within a cell are functionally constrained, it is possible to encode the same polypeptide with multiple different nucleotide sequences by using different synonymous codons. This redundancy in the genetic code, coupled with the difference in nucleotide nitrogen content, means that it is possible to reduce the allocation of cellular resources to transcript sequences without altering protein sequence or presumed function (Seward and Kelly 2017). For example, a single A to T substitution in a highly expressed transcript such as RuBisCo small subunit (∼5,000 transcripts per cell) saves an equivalent amount of nitrogen (∼15,000 atoms) as is contained in three complete RuBisCo holoenzymes (∼5,000 nitrogen atoms per hexadecamer). It was thus hypothesized that plants that require increased quantities of nitrogen to fix CO_2_ would be more nitrogen limited and thus natural selection would favor codons in transcript sequences that required less nitrogen to biosynthesize.

Here, it is shown that plants that require more nitrogen to conduct photosynthesis experience stronger selection to minimize transcript nitrogen biosynthesis cost. Furthermore, it is demonstrated that the strength of selection acting on transcript sequence biosynthesis cost explains a significant proportion of variation in gene evolutionary rate, whereby genes that experience stronger selection to minimize cost are evolving slower than genes that experience weaker selection. Together these findings directly link photosynthetic efficiency of a plant to the rate at which its genes and genome evolve, and provide a mechanistic link between fluctuation in rates of plant diversification and changing environmental conditions.

## Results

### Variation in Photosynthetic Nitrogen Use Efficiency Modulates the Strength of Selection Acting on Transcript Nitrogen Cost

**Fig. 1.**
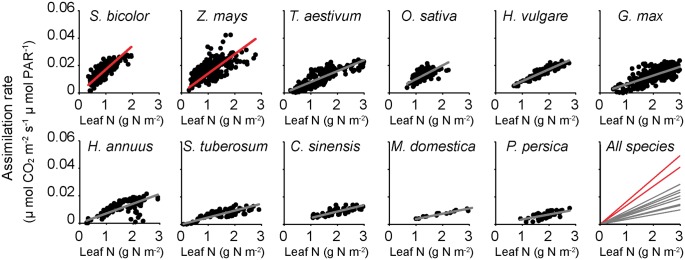
Scatter plots depicting the relationship between light saturated photosynthetic rate and leaf nitrogen content for the 11 species used in this analysis. A higher resolution plot with *R*^2^ values and slopes is provided as supplementary file S1, Supplementary Material online. Light saturated CO_2_ assimilation rate (µ mol CO_2_ m^−2 ^s^−1 ^µ mol PAR^−1^) and leaf nitrogen (g N m^−2^). Each fitted line is also provided on the plot labeled “All Species” to make it easier to compare the range of relationships that are observed between light saturated CO_2_ assimilation rate and leaf nitrogen. Fitted lines for C_4_ species are shown in red and fitted lines for C_3_ species shown in gray. The slope of each fitted line is the photosynthetic nitrogen use efficiency (PNUE) for each species. *Sorghum bicolor* (Muchow and Sinclair 1994; Anten et al. 1995; Tominaga et al. 2015), *Zea mays* (Muchow and Sinclair 1994; Lindquist and Mortensen 1999; Osaki et al. 2001; Paponov and Engels 2003; Drouet and Bonhomme 2004; Paponov et al. 2005; Vos et al. 2005), *Triticum aestivum* (Evans 1983; Fischer et al. 1998; Osaki et al. 2001; Müller et al. 2005), *Oryza sativa* (Anten et al. 1995; Osaki et al. 2001; Ohsumi et al. 2007; Hirasawa et al. 2010), *Hordeum vulgare* (Braune et al. 2009), *Glycine max* (Anten et al. 1995; Osaki et al. 2001; Maekawa and Kokubun 2005; Rotundo and Borras 2016), *Helianthus annuus* (Fredeen et al. 1991; Andrade et al. 1993; Gimenez et al. 1994; Trápani and Hall 1996; Bange et al. 1997; Rodríguez et al. 1998), *Solanum tuberosum* (Vos and van der Putten 1998, 2001), *Citrus sinensis* (Romero-Aranda and Syvertsen 1996), *Malus domestica* (Cheng and Fuchigami 2000), and *Prunus persica* (DeJong et al. 1989; Rosati et al. 1999; Malcolm et al. 2008).

**Fig. 2.**
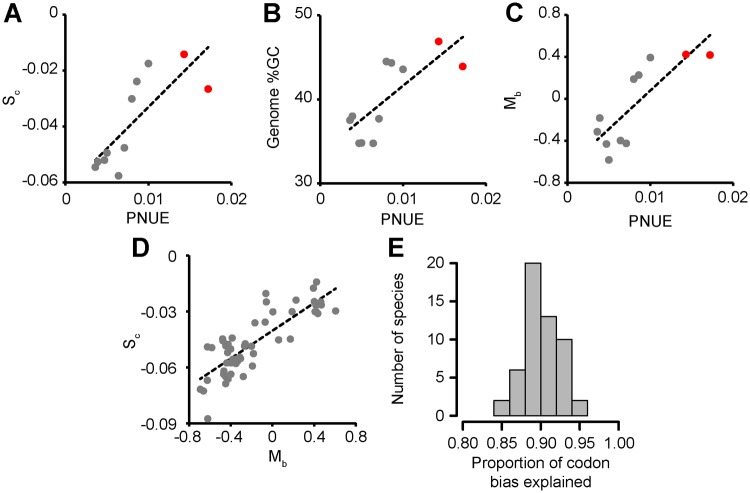
Photosynthetic nitrogen use efficiency (PNUE) modulates selection acting on transcript biosynthesis cost and mutation bias. Plots in parts A to C depict the same species set. C_4_ species are shown as red points and C_3_ species are shown as gray points. Complete data sets are provided in supplementary file S1, Supplementary Material online. (*A*) The relationship between PNUE and the strength of selection acting on transcript biosynthesis cost (*S*_c_, *R*^2^ = 0.62) for these species. (*B*) The relationship between PNUE and the Genome wide GC content (*R*^2^ = 0.58). (*C*) The relationship between PNUE and mutation bias acting on coding sequences here values >0 indicate mutation bias toward GC and values <0 indicate a mutation bias toward AT (*M*_b_, *R*^2^ = 0.65). (*D*) The relationship between *S*_c_ and *M*_b_ for all angiosperm species in Phytozome (*R*^2^ = 0.69). (*E*) The proportion of codon bias that can be explained by the joint effects of mutation bias and selection acting on nitrogen biosynthesis cost.

To test the hypothesis that variation in the amount of nitrogen used for photosynthesis influences the strength of selection acting on transcript biosynthesis cost, an analysis of molecular sequence evolution was conducted for 11 plant species for which both whole genome sequences (Goodstein et al. 2012) and accurate photosynthetic nitrogen use efficiencies (Rotundo and Cipriotti 2017) were available. This set of species included both C_3_ and C_4_ grasses, as well as C_3_ herbs and trees encompassing a broad range of PNUE values (fig. 1 and supplementary table S1 sheet 1, Supplementary Material online). For each species, the strength of selection acting on transcript nitrogen cost [*S*_c_] was inferred from the complete set of open reading frames in the respective genome using the SK model (Seward and Kelly 2016) implemented using CodonMuSe (Seward and Kelly 2017). Consistent with the hypothesis, those species that required more nitrogen to conduct photosynthesis exhibited stronger selection (more negative value for *S*_c_) to minimize the nitrogen biosynthesis cost of transcript sequences (*R*^2^ = 0.62, *P* < 0.001, fig. 2*A*). However, both PNUE and *S*_c_ exhibited significant phylogenetic signal (supplementary file S2, Supplementary Material online), and thus a phylogenetic least squares (PGLS) analysis was conducted to account for this effect. This PGLS approach makes the implicit assumption that traits evolve similarly across the phylogeny (Keck et al. 2016). This assumption is invalid for evolutionary transitions from C_3_ to C_4_ photosynthesis, as this change is concomitant with a rapid change in PNUE that is disproportionate to phylogenetic distance. Similarly, this assumption is also invalid for the evolution of root-nitrogen fixation as legumes export photosynthate for use in nitrogen fixation and thus the amount of carbon acquired per unit nitrogen in the leaf is an overestimate of the amount of carbon acquired by the plant. Thus, all PGLS model were built using data from the C_3_ species (supplementary file S2, Supplementary Material online). Correction for phylogenetic signal did not remove the significant positive association between PNUE and S_c_ (*R*^2^ = 0.78, *P* = 0.004, supplementary file S2, Supplementary Material online) and thus the amount of nitrogen used for photosynthesis in a plant modulates the strength of selection acting on its transcript nitrogen biosynthesis cost.

It has previously been shown that the strength of selection acting on transcript biosynthesis cost and translational efficiency acts in proportion to the mRNA abundance of a gene (Seward and Kelly 2016, 2017). Comparison of gene-wise estimates for *S*_c_ and *S*_t_ with mRNA abundance estimates obtained from whole-plant RNA-Seq in *Arabidopsis thaliana* revealed that the same phenomenon also occurs in plants (supplementary file S3, Supplementary Material online). Thus, the magnitude of selection acting on an individual gene is modulated by both the abundance of the mRNA and the amount of nitrogen used for photosynthesis.

### Variation in Photosynthetic Nitrogen Use Efficiency Influences Variation in Mutation Bias and Genome-Wide GC Content

As the nitrogen biosynthesis cost of DNA sequences varies (AT pairs require 7 and GC pairs require 8 nitrogen atoms), it was further hypothesized that those species that required more nitrogen to conduct photosynthesis would exhibit a stronger genome-wide mutation bias toward AT base pairs. Consistent with the hypothesis, those species that required more nitrogen to conduct photosynthesis had lower genome-wide GC content and thus invested less nitrogen in their genome sequences (*R*^2^ = 0.58, *P* < 0.001, fig. 2*B*). This phenomenon was also apparent from the analysis of coding sequences, where codon mutation bias toward AT rich codons was stronger in species that had lower photosynthetic nitrogen use efficiencies (*R*^2^ = 0.65, *P* < 0.001, fig. 2*C*). Like for *S*_c_, both genome-wide GC content and mutation bias exhibited significant phylogenetic signal (supplementary file S2, Supplementary Material online). However, in contrast to the case for *S*_c_ correction for phylogenetic signal reduced the strength of the positive association with PNUE in C_3_ species such that they failed to achieve statistical significance (*P* ≥ 0.05, supplementary file S2, Supplementary Material online).

**Fig. 3.**
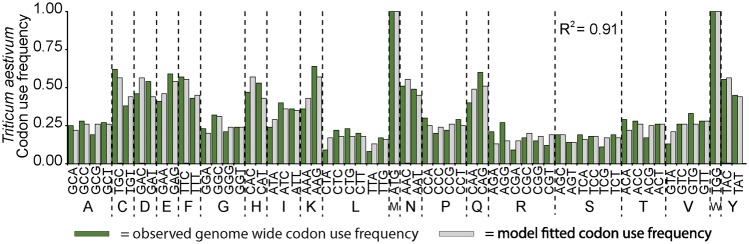
A comparison of model-fitted and genome wide patterns of synonymous codon use. Example shown is from *Triticum aestivum*. The calculation of the coefficient of determination (*R*^2^) did not include amino acids with only one codon (M or W). *T. aestivum* was chosen as it is close to the average *R*^2^ value of the species analyzed. The complete set of plots can be found in supplementary file S3, Supplementary Material online.

To exclude the possibility that low sample size caused the statistical test to fail, an additional analysis on a larger species set was conducted. If PNUE influences genome-wide GC content and mutation bias, then there should be a dependency between *S*_c_ and these traits in C_3_ species from across the angiosperm phylogenetic tree. However, if there is no association between GC content, mutation bias and PNUE then *S*_c_ will also be independent of GC content and mutation bias. To investigate this, a larger set of C_3_ angiosperm genomes on Phytozome were analyzed to determine whether there was a global, significant, positive association between *S*_c_ and GC content and mutation bias. As postulated, those C_3_ species that exhibited stronger selection acting on transcript biosynthesis cost also exhibited lower genome-wide GC content (*R*^2^ = 0.69, fig. 2*D* and supplementary table S1 sheet 2, Supplementary Material online). Correcting for phylogenetic signal did not remove the significant positive association (*R*^2^ = 0.21, *P* = 0.007, supplementary file S2, Supplementary Material online). An analogous result was also obtained if estimates of mutation bias were obtained from genome sequence with no current gene annotation (supplementary file S2, Supplementary Material online). Therefore, the most parsimonious explanation is that variance in PNUE is a determinant of biased patterns of nucleotide use in both genome and transcriptome sequences. Furthermore, when mutation bias (as calculated by the SK model) and selection acting on transcript nitrogen cost are considered together, they are sufficient to explain ∼90% of variance in genome-wide patterns of synonymous codon use in all plant species tested (fig. 2*E* and supplementary table S1 sheet 2, Supplementary Material online). To illustrate this explanatory power an example codon usage plot and model fit is provided for *Triticum aestivum* in figure 3. Analogous plots for each of the other eleven species are provided in supplementary file S4, Supplementary Material online. Other factors not included in this analysis account for the remaining unexplained variation in synonymous codons use.

### Variation in Photosynthetic Nitrogen Use Efficiency Influences Variation in Nitrogen Content of Amino Acid Side Chains in Conserved Basic Sites

**Fig. 4.**
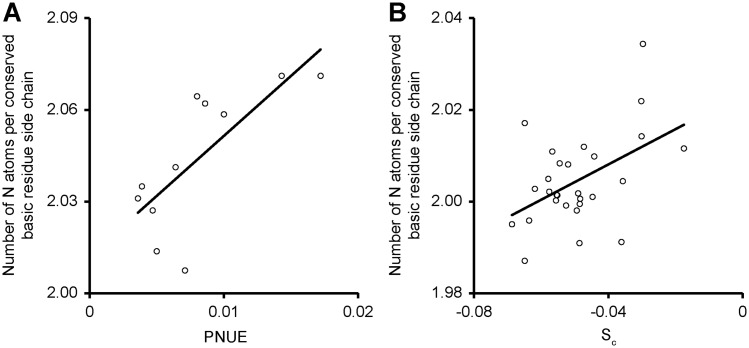
PNUE modulates the amino acid use at conserved basic sites in protein sequences. (*A*) The relationship between PNUE and the number of nitrogen atoms in side chains of ungapped, conserved, basic sites in single copy orthologous genes (*R*^2^ = 0.56, *P *= 0.008). (*B*) The relationship between *S*_c_ the number of nitrogen atoms in side chains of ungapped, conserved, basic sites in orthologous genes (*R*^2^ = 0.23, *P* = 0.01).

It has previously been shown that nitrogen limitation can cause a reduction in the nitrogen content of proteins in marine (Grzymski and Dussaq 2012) and parasitic microorganisms (Seward and Kelly 2016). Given the observed interaction between PNUE and the strength of selection acting on transcript biosynthesis cost, it was investigated whether a similar effect could be detected in the nitrogen content of amino acid side chains. Most amino acids used in construction of proteins contain a single nitrogen atom. However, six of the 20 also contain one or more nitrogen atoms in their side chains (R = 4 nitrogen atoms, H = 3, K = 2, N = 2, Q = 2, W = 2). Although, analogous redundancy to the codon code does not exist for amino acids, some amino acids exhibit similar functional properties. Of the 6 amino acids with nitrogen atoms in their side chains, three (R, H, and K) have basic side chains at neutral pH and thus could be considered to exhibit some biochemical redundancy with each other. Moreover, these three basic residues vary in their nitrogen content. Therefore, to determine whether variation in PNUE caused a concomitant variation in the nitrogen content of protein sequences, an analysis was conducted on 2,545 ungapped aligned basic sites in 124 ubiquitously conserved single copy genes in the 11 species. Here, ungapped sites that contained only basic residues were analyzed so it could be assumed that there is a functional constraint on the biochemical properties of the residue present, and that to some extent basic resides may be able to act redundantly at these positions. Consistent with the analysis of transcript sequences and genome GC content, those species that required more nitrogen to conduct photosynthesis contained fewer nitrogen atoms in amino acid side chains at conserved basic sites in ubiquitously conserved genes (*R*^2^ = 0.55, *P* = 0.008, fig. 4*A*). However, correction for phylogenetic signal reduced the strength of the positive association with PNUE such that it failed to achieve statistical significance for the C_3_ species within this group (*P* ≥ 0.05).

As before, to exclude the possibility that low sample size caused the statistical test to fail, an additional analysis on a larger set of species was conducted. If PNUE influences nitrogen content in amino acid sequences, then there should be a dependency between *S*_c_ and amino acid nitrogen content at conserved basic sites across the angiosperm phylogenetic tree. As above, a larger set of C_3_ species was analyzed and those species that exhibited stronger selection acting on transcript biosynthesis cost also exhibited lower nitrogen content at conserved basic sites (*R*^2^ = 0.23, *P* = 0.01, fig. 4*B*). Correcting for phylogenetic signal did not remove the significant positive association (*R*^2^ = 0.14, *P* = 0.026). Therefore, the most parsimonious explanation is that variance in PNUE also influences patterns of amino acid use in protein sequences.

### Variation in the Strength of Selection Acting on Nitrogen Biosynthesis Cost Contributes to Variation in Gene Evolutionary Rate

Given that variance in PNUE is associated with variance in the strength of selection acting on gene sequences, it was postulated that this would cause a concomitant variance in molecular evolutionary rate of genes. Specifically, those genes that experience stronger selection to minimize transcript nitrogen cost would have lower rates of molecular evolution when compared with genes that experience weaker selection. This phenomenon occurs because spontaneous mutations that increase transcript biosynthesis cost will be more deleterious in genes that experience stronger selection to minimize cost irrespective of whether that mutation is synonymous or nonsynonymous (Seward and Kelly 2017). As mutations that are more deleterious will be lost more rapidly, this results in a lower molecular evolution rate for genes that experience stronger selection (Seward and Kelly 2017). This phenomenon has previously been observed for bacterial genes (Seward and Kelly 2017).

**Fig. 5.**
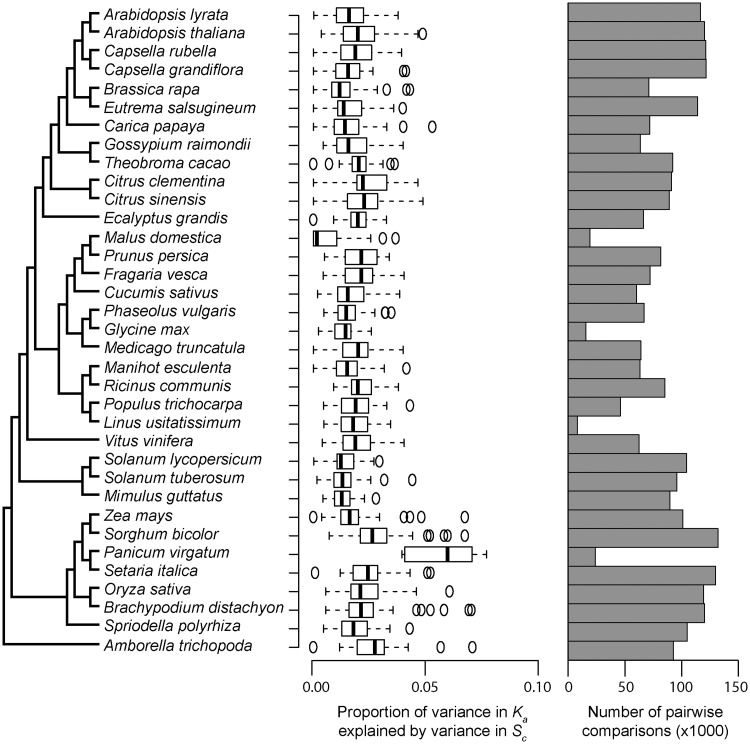
The strength of selection acting on transcript biosynthesis cost constrains the rate of amino acid sequence evolution. Gray bars depict mean values estimated from all possible pairwise comparisons featuring the species under consideration. Phylogenetic tree adapted from Phytozome (Goodstein et al. 2012).

To investigate this, both the number of synonymous substitutions per synonymous site (*K*_s_) and the number of nonsynonymous substitutions per nonsynonymous site (*K*_a_) were estimated from pairwise alignments of single copy orthologous genes in a set of 38 plant species (fig. 5). The strength of selection acting on transcript nitrogen cost was also inferred for each individual gene using CodonMuSe (Seward and Kelly 2017). For each species pair, these data were subject to multiple regression analysis to estimate the proportion of variance in *K*_a_ or *K*_s_ that was explained by variance in *S*_c_ between that species pair (supplementary file S2 and table S1 sheet 3, Supplementary Material online). Consistent with the hypothesis, genes experienced stronger selection to minimize transcript nitrogen cost evolved more slowly than those that experience weaker selection (fig. 5 and supplementary file S2, Supplementary Material online). Moreover, variance in the strength of selection explained up to 10% of variance in synonymous site evolutionary rate (supplementary file S2, Supplementary Material online) and ∼2% of variance in nonsynonymous site evolutionary rate across all species (fig. 5 and supplementary file S2, Supplementary Material online). Thus, as genome-wide values for *S*_c_ are linked to PNUE, it follows that the tempo of the plant molecular clock is modulated by changes in PNUE.

## Discussion

There is substantial interspecies variation in the amount of nitrogen required to conduct photosynthesis in plants (Evans 1989; Rotundo and Cipriotti 2017). In this work, it is shown that this variation is a determinant of plant gene and genome composition, and modulates the rate at which plant gene sequences evolve. The findings presented here provide significant new insight into the relationship between metabolism, the environment, and molecular evolution in plants. They are also compatible with previous reports that revealed that wild plants contained less nitrogen in their DNA when compared with domesticated relatives that had been supplemented with nitrogen fertilizer for thousands for years (Acquisti et al. 2009).

Multiple factors have previously been proposed to bias the relative use of synonymous codons. These include but are not limited to; mutational biases during DNA replication and repair (Eyre-Walker 1991; Francino and Ochman 1999; Rao et al. 2011), selection due to difference in abundance of iso-accepting tRNAs (Plotkin et al. 2004), selection to modulate translational efficiency and accuracy (Sorensen et al. 1989; Akashi 1994; Shah and Gilchrist 2011), selection acting on altered gene splicing and protein folding (Shah and Gilchrist 2011), selection on RNA secondary structure (Vandivier et al. 2016), transcription-associated mutation bias (Comeron 2004), mRNA purine loading as a result of growth in high temperature (Lao and Forsdyke 2000; Paz et al. 2004), selection for certain dinucleotides and trinucleotides (Camiolo et al. 2015). The results presented here do not preclude these effects but rather build upon our understanding of factors affecting the relative use of synonymous codons.

Speciation and extinction rates in plants are a function of molecular substitution rate, such that lineages with higher rates of molecular substitution have higher rates of speciation and extinction (Lancaster 2010). Therefore, the mechanistic link between PNUE and molecular evolution presented here has significant implications for our understanding of the past, present, and future of plant evolution. For example, plants with higher PNUE, and thus with higher rates of molecular evolution, will have therefore higher rates of speciation and extinction. As a corollary, evolutionary adaptations that increase PNUE will also increase rates of speciation and extinction. For example, the suite of molecular and anatomical changes that facilitate the evolution C_4_ photosynthesis result in a dramatic reduction in the amount of nitrogen required to conduct photosynthesis. The findings presented here predict that this increase in PNUE would cause a concomitant reduction in the strength of selection on gene sequences and therefore result in an increased rate of molecular evolution. Thus, PNUE-driven increase in molecular evolution rate provides a simple mechanistic explanation for the increase in rates of speciation that are observed concomitant with the evolution of C_4_ photosynthesis (Spriggs et al. 2014).

**Fig. 6.**
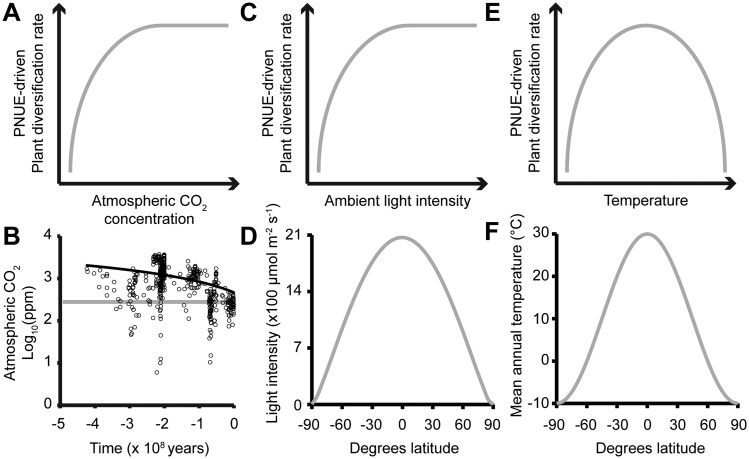
Photosynthetic nitrogen use efficiency (PNUE) modulates plant diversification. (*A*) Cartoon depicting the proposed relationship between PNUE-driven plant diversification and atmospheric CO_2_ concentration. (*B*) Changes in atmospheric CO_2_ concentration (ppm = parts per million) over the last 500 My, least squares linear fit shown as solid black line. Gray horizontal line indicates preindustrial CO_2_ levels (278 ppm) adapted from Foster et al. (2017). (*C*) Cartoon depicting the relationship between PNUE and light intensity. (*D*) The photon flux density on the surface of the earth at 0° longitude at noon on the vernal equinox. (*E*) Cartoon depicting the proposed relationship between PNUE-driven plant diversification and ambient temperature. (*F*) The average (contemporary) annual temperature at the surface of the earth (Randel et al. 2004).

Increases in atmospheric CO_2_ concentration cause corresponding increases in PNUE in plants. In the short term, this increase in PNUE is caused by a reduction in the rate of photorespiration (Chollet and Ogren 1975). In the long term, plants also adapt to higher CO_2_ concentration by reduction in the investment of cellular resources in photosynthesis protein production (Stitt and Krapp 1999). Thus, when atmospheric CO_2_ increases, PNUE increases. The link between PNUE and molecular evolution presented here predicts that this increase in PNUE will cause a corresponding increase in molecular evolution rate, and thus an increase in the rate of plant diversification (fig. 6*A* and *B*). This therefore provides a mechanistic explanation for the observed relationship between plant diversification rates observed in the fossil record and changes in atmospheric CO_2_ concentration (McElwain et al. 2011).

Similar to changes in CO_2_ availability, changes in other environmental factors such as light availability (fig. 6*C* and *D*) and temperature (fig. 6*E* and *F*) also influence photosynthetic rate and thus PNUE. Unlike CO_2_, these other environmental factors vary widely over the surface of the planet. For example, light intensity and temperature are not uniformly distributed on the surface of the earth, but instead decrease as a function of distance from the equator (fig. 6*D* and *F*). This variation is due to the curvature of the earth and the corresponding increase in the angle of the incident light. The findings presented here predict that plant diversification rates will be higher toward the equator where light and temperature are less limiting on photosynthesis and thus PNUE will be higher. These findings therefore provide additional insight into the plant species latitude diversity gradient (Mittelbach et al. 2007; Gillman and Wright 2014), where rates of plant diversification are higher in regions that are closer to the equator.

It has previously been shown that fossil plant genome size exhibits a strong positive correlation with atmospheric CO_2_ concentration (Franks et al. 2012). Given that guard cell volume is strongly linked to genome size, it was proposed that selection acting on guard cell volume adapted the aperture of stomata for different atmospheric CO_2_ concentrations (Franks et al. 2012). The findings presented here may provide additional mechanistic insight into this phenomenon. Specifically, increases in atmospheric CO_2_ concentration cause increases in PNUE. This increase in PNUE causes a concomitant reduction in the strength of selection to minimize resource allocation to transcript sequences, genome sequences (assessed in this work by changes in mutation bias and GC content) and protein sequences (assessed in this work by changes in nitrogen content of amino acid side chains). It follows that this reduction in selection likely also applies to genome size, such that increases in atmospheric CO_2_ facilitate concomitant increases in genome size via reduction in selection to minimize resource allocation to DNA. Therefore, changes in PNUE provide an additional mechanistic explanation for the relationship between fossil plant genome size and atmospheric CO_2_ concentration. It should be noted here that molecular sequence analysis was not conducted as the genome sequences for these fossil plant species no longer exist.

## Conclusion

Plants build their genes and genomes from monomers assembled from inorganic carbon and nitrogen. Of these two, nitrogen is more limiting such that plants that require higher quantities of nitrogen to conduct photosynthesis have less nitrogen available for other uses and thus experience stronger selection to reduce nitrogen investment in gene sequences. A multitude of environmental factors can exacerbate or ameliorate PNUE. Therefore, both the environment and genetic factors can modulate the strength of selection acting to reduce nitrogen investment in gene sequences and hence modulate plant genome composition and molecular evolution. Hence, at multiple scales plant evolution is modulated by the amount of nitrogen required to conduct photosynthesis.

## Materials and Methods

### Data Sources

The genome sequences and corresponding set of representative gene models for each species were downloaded from Phytozome V12 (Goodstein et al. 2012). The *Helianthus annuus* genome was obtained from (Badouin et al. 2017). Photosynthetic measurements and leaf nitrogen measurements were obtained from Rotundo and Cipriotti (2017).

### Inference of Selection Acting on Codon Usage Bias

To obtain the number of tRNA genes in each genome, tRNAscan (Lowe and Eddy 1997) was run on each of the plant genomes. For each species the tRNAscan output file and the complete set of representative coding sequences was analyzed using CodonMuSe (Seward and Kelly 2017). This provided the values for mutation bias (*M*_b_) as well as the composite parameters of selection acting on transcript biosynthesis cost (*S*_c_), and selection acting on translational efficiency (*S*_t_) for each species in this analysis. CodonMuSe by default estimates the proportion of variance in codon use that can be explained by the mutation bias and these selective forces.

### Phylogenetic Tree Inference for the 11 Species with PNUE Data

The complete set of proteomes for the 11 species used in this analysis was subject to orthogroup inference using OrthoFinder (Emms and Kelly 2015). In the case of hexaploid wheat genome, only proteins derived from genes present in the wheat A genome were used for orthogroup inference. Orthogroups containing proteins derived from single copy genes in each of the 11 species were selected and aligned using the MAFFT (Katoh and Standley 2016) L-INS-i algorithm. These alignments were trimmed to remove any columns containing gap characters and then concatenated to form a multiple sequence alignment containing 4,949 aligned amino acid positions in each species. This alignment and was subject to bootstrapped maximum likelihood phylogenetic tree inference using IQ-TREE (Nguyen et al. 2015) while estimating the best fitting model of sequence evolution from the data. The best fitting model was inferred to be JTTDCMut + F+G4 by Bayesian information criterion. This tree was used for the phylogenetic least squares analysis and is provided in supplementary file S2, Supplementary Material online.

### *K*_a_ *and K*_s_ Estimation and Comparison with *S*_c_

The predicted proteins from 38 species were downloaded from Phytozome. These species were subject to orthogroup and ortholog inference using OrthoFinder (Emms and Kelly 2015). All 1,406 pairwise comparisons between species were subsequently conducted. Each pairwise comparison comprised the following steps. 1) The full set of single copy orthologs for the species pair under consideration were isolated. 2) The protein sequences for each orthologous pair were aligned using MAFFT (Katoh et al. 2005) L-INS-i and the coding sequences rethreaded back through the protein sequence alignment. 3) The resulting coding sequence alignments were parsed to remove any gap-containing columns. 4) Ungapped alignments containing >100 aligned codons were subject to *K*_a_ and *K*_s_ inference using KaKsCalculator v2.0 (Wang et al. 2010) using the default settings. Additional data filtering and quality control were carried out as described in supplementary file S2, Supplementary Material online. Individual estimates for *S*_c_ and *S*_t_ were obtained for each gene in the 38 species using CodonMuSe. Here, the value for *M*_b_ in each inference was set to the genome-wide value estimated from an analysis of all genes. Pairwise species comparisons that had > 100 genes satisfying all filtration criteria were selected for further analysis.

The value for *K*_a_ and *K*_s_ are dependent on several factors:

(1) $$K_{x}=f\left(T_{d1},{T_{d2,}S}_{1},S_{2},M_{1},M_{2},N_{e_{1}},N_{e_{2}}\right),$$

where *K*_x_ is either *K*_a_ or *K*_s_, *T*_d1_ is the divergence time in number of generations between species 1 and the most recent common ancestor of the species pair being analyzed, *S*_1_ is the strength of selection acting on the sequence of the gene in species 1, *M*_1_ is the mutation rate species 1, and *N*_e1_ is the effective population size of species 1. *S*_c_ is a composite parameter (Seward and Kelly 2016) that is a product of a component of the selection coefficient *S*_1_ and the effective population size *N*_e1_. Thus, each pairwise species comparison was subject to multiple regression analysis using the lm function in R using the following model:

$$\ln K_{x}=\beta_{1}S_{c_{1}}+\beta_{2}S_{c_{2}},$$

where *β*_1_ thus incorporates both *T*_d1_ and *M*_1_. Thus, the multiple regression evaluates the component of variance in *K*_a_ or *K*_s_ that is attributable to both *S*_c1_ and *S*_c2_. The natural log of the *K*_a_*and K*_s_ estimates were taken as both *K*_a_ and *K*_s_ are log-normally distributed whereas *S*_c_ is normally distributed. All data were confirmed to be normally distributed by the Shapiro–Wilks test for normality prior to use in regression analysis. The mean of the adjusted *R*^2^ for all pairwise comparisons featuring a given species was taken as an estimate the proportion of variance that is explained by variation in *S*_c_ for that species.

### Quantification of mRNA Abundance

To provide whole-organism mRNA abundance estimates the NCBI SRA database was searched for RNA-Seq samples from whole plants. A single experiment containing three biological replicates of whole-plant RNA-Seq from *Arabidopsis thaliana* 8 day old seedlings was obtained from BioProject PRJNA384979 (Major et al. 2017). The raw reads were downloaded, subject to quality filtering using trimmomatic (Bolger et al. 2014). This was done to remove low quality bases and read-pairs as well as contaminating adaptor sequences prior to quantification. Sequences were searched for all common Illumina adaptors (the default option) and the settings used for read processing by trimmomatic were LEADING: 10 TRAILING: 10 SLIDINGWINDOW: 5: 15 MINLEN: 25. Following trimming, the processed reads were subject to quantification estimation using the complete set of transcript sequences for protein coding genes *A. thaliana* from Phytozome v12 using Salmon v0.9.1 (Patro et al. 2017) with the –seqBias option enabled. TPM values for multiple transcript variants were summed so that a single TPM estimate was provided for each gene for each biological replicate. The mean TPM value of the three biological replicates was taken as the abundance estimate for that gene. Transcripts with mean TPM values ≥ 1 were selected for analysis.

### Analysis of Amino Acid Side Chains

Two data sets were constructed to analyze the effect of variation in PNUE on amino acid nitrogen content. The first focused on single copy orthologous genes. Here, the 11 species with PNUE data were subject to orthogroup inference using OrthoFinder (Emms and Kelly 2015). Orthogroups containing only single copy genes (*N* = 130) were identified. The amino acid sequences were aligned using MAFFT L-INS-I (Katoh and Standley 2014) and ungapped aligned positions containing only basic residues (R, H, and K) were selected for further analysis. Of the 130,124 alignments contained ungapped basic positions comprising a total of 2,545 aligned positions. The mean number of *N* atoms per ungapped aligned position was calculated as the mean of these 2,545 aligned positions.

An equivalent set of single copy orthologous genes was not available for the larger species analysis. This was because of gene duplication and loss which meant that there were no orthogroups present as a single copy gene in all species under consideration. Thus an analogous analysis was performed. Here, the amino acid sequences from orthogroups containing all species were aligned (*N* = 4,996). Of these alignments, 1,098 contained ungapped basic positions found in at least one representative sequence from all species with a mean number of 11,835 sites per species. The mean number of *N* atoms per ungapped aligned position was calculated as the mean of these sites.

## Supplementary Material

Supplementary data are available at *Molecular Biology and Evolution* online.

## Supplementary Material

Supplementary Data
