# Supplementary material for: The Amount of Nitrogen Used for Photosynthesis Modulates Molecular Evolution in Plants
Source: Mol Biol Evol. 2018 Apr 19;35(7):1616–25. doi: 10.1093/molbev/msy043 (PMC5995192; doi:10.1093/molbev/msy043)
Supplement: Supplementary Data [file msy043_supp.zip › Supplemental_File_S2.pdf]

Accounting for phylogenetic signal in comparisons of PNUE

The amount of phylogenetic signal in each individual trait was estimated using the phylosignal R package[1]. Five distinct methods were implemented for estimating the extent and significance of the phylogenetic signal, including Abouheif's C_{mean}, Blomberg's K, Blomberg's K*, Moran's I, and Pagel's Lambda[1]. Each individual trait under consideration in this analysis was interrogated in the context of a maximum likelihood phylogenetic tree for the 11 species. Photosynthetic nitrogen use efficiency (PNUE), Selection acting on biosynthesis costs (S_c), mutation bias (Mb), and genome-wide GC content (GC) all showed significant evidence for phylogenetic signal (Table 1). Thus, as expected more closely related species have more similar PNUE, more similar GC content and more similar strength of selection acting on transcript biosynthesis cost than more distantly related species.

To correct for the influence of the underlying phylogeny in the comparisons presented in Fig. 1 of the main text, phylogenetic generalised least squares (PGLS) was used[2] as implemented in the R package caper[3]. This PGLS approach makes the implicit assumption that traits evolve similarly across the phylogeny[1]. This assumption is invalid for evolutionary transitions from C_3 to C_4 photosynthesis, as this change is concomitant with a rapid change in PNUE that is disproportionate to phylogenetic distance. Similarly, this assumption is also invalid for the evolution of root-nitrogen fixation, because legumes export photosynthate for use in nitrogen fixation and thus the amount of carbon acquired per unit nitrogen in the leaf is an overestimate of the amount of carbon acquired by the plant (when compared to non-nitrogen-fixing plants). Thus, the PGLS model was built using data only the C_3 species. Three PGLS models were constructed 1) Sc against PNUE, 2) Mb against PNUE and 3) GC against PNUE. In each case, the maximum likelihood value of Pagel's λ estimated from the data was 1. Thus the most likely model assumed an underlying tree that was identical to the species tree and that there was phylogenetic signal in the data. Moreover, as Pagel's λ is equal to 1, the results of the PGLS are identical to those that would be obtained via Felsenstein's phylogenetic independent contrasts[4].

For the C_3 species alone, the R^2 between PNUE and S_c was 0.90. After correcting for phylogeny the R^2 between PNUE and *Sc* decreased to 0.78 ($p = 0.004$). Thus, PNUE of a C_3 plant determines the strength of selection acting on transcript biosynthesis cost and it is possible to predict the strength

of that selection coefficient from knowledge of a C_3 plant's photosynthetic nitrogen use efficiency with ~78% accuracy. In contrast, accounting for phylogeny reduced the strength of the relationship between PNUE and GC, and PNUE and M_b. Here the coefficients of determination decreased from 0.66 to 0.23 ($p \leq 0.23$), and from 0.62 to 0.39 ($p \leq 0.1$) respectively. Thus, the association between genome wide GC content and PNUE fails to pass a significance threshold when phylogeny is taken into consideration.

R code for PGLS implementation

```
library("ape")
library("caper")
data<-read.table("data_for_R.txt", header=TRUE)
c3data <- data[-8:-9,]
c3data <- c3data[-2,]
tree<-read.tree("rooted_tree.tree")
c3tree<-drop.tip(tree, "Sorghum", trim.internal = TRUE, subtree = FALSE, root.edge = 0, rooted = is.rooted(tree))
c3tree<-drop.tip(c3tree, "Maize", trim.internal = TRUE, subtree = FALSE, root.edge = 0, rooted = is.rooted(tree))
c3tree<-drop.tip(c3tree, "Soybean", trim.internal = TRUE, subtree = FALSE, root.edge = 0, rooted = is.rooted(tree))
PGLS <-(comparative.data(phy = c3tree, data = c3data, names.col = Species, na.omit = FALSE, vcv = TRUE, warn.dropped = TRUE))
modelSc.pgls<-pgls(Sc ~ PNUE, data = PGLS, lambda='ML')
modelGC.pgls<-pgls(GC ~ PNUE, data = PGLS, lambda='ML')
modelMbCDS.pgls<-pgls(MbCDS ~ PNUE, data = PGLS, lambda='ML')
modelMbGenome.pgls<-pgls(MbGenome ~ PNUE, data = PGLS, lambda='ML')
modelAA.pgls<-pgls(AA ~ PNUE, data = PGLS, lambda='ML')
summary(modelSc.pgls)
summary(modelGC.pgls)
summary(modelMbCDS.pgls)
summary(modelMbGenome.pgls)
summary(modelAA.pgls)
data$predictedSc <- predict.pgls(modelSc.pgls, data)
data$predictedGC <- predict.pgls(modelGC.pgls, data)
data$predictedMbCDS <- predict.pgls(modelMbCDS.pgls, data)
data$predictedMbGenome <- predict.pgls(modelMbGenome.pgls, data)
data$predictedAA <- predict.pgls(modelAA.pgls, data)
write.table(data, file="new_data_file_with_predictions_from_C3_PNUE_model.txt")
```

Data matrix for input to PGLS

Species	PNUE	Mb	Sc	GC	AA
Grapefruit	0.0047	-0.4305	-0.052	34.77	2.0271
Soybean	0.0064	-0.3979	-0.0576	34.76	2.0413

Sunflower	0.0071	-0.4253	-0.0476	37.69	2.0075
Potato	0.005	-0.5824	-0.0494	34.81	2.0138
Barley	0.0086	0.2258	-0.0239	44.34	2.0621
Wheat	0.008	0.1875	-0.0301	44.5	2.0644
Rice	0.01	0.3925	-0.0175	43.57	2.0585
Sorghum	0.0172	0.417	-0.0266	43.91	2.0711
Maize	0.0143	0.4226	-0.0142	46.89	2.0711
Apple	0.0039	-0.1829	-0.0526	37.99	2.0350
Peach	0.0036	-0.3138	-0.0545	37.51	2.0310

Phylogenetic tree for input to PGLS

(((Sunflower:0.095882,Potato:0.113035):0.018436,(Soybean:0.109306,(Grapefruit:0.086121,(Apple:0.04533,Peach:0.029469):0.052594):0.01232):0.017255):0.06550932,(((Barley:0.008,Wheat:0.01424):0.043615,Rice:0.047131):0.015232,(Sorghum:0.014111,Maize:0.022183):0.033334):0.12565468);

Table 1

Table 1. Results of phylogenetic signal tests for traits analysed in this study

	C_{mean}	p-value	I	p-value	K	p-value	K*	p-value	λ	p-value
**PNUE**	0.51	0.005	0.31	0.002	1.26	0.002	1.18	0.003	0.93	0.017
**S_c**	0.55	0.003	0.35	0.004	2.1	0.002	1.73	0.003	0.89	0.001
**GC**	0.51	0.005	0.36	0.003	2.73	0.002	2.23	0.003	0.99	0.001
**M_b**	0.52	0.003	0.34	0.004	3.37	0.001	2.92	0.001	NA	NA

Accounting for phylogenetic signal in larger multispecies comparisons

To exclude the possibility that low sample size caused the statistical association between either PNUE and mutation bias or PNUE and GC content to fail, an additional analysis on a larger species set was conducted. If PNUE influences genome-wide GC content and mutation bias, then there should be a dependency between S_c and these traits when larger numbers of species are considered. However, if there is no association between GC content, mutation bias and PNUE then S_c will also be independent of GC content and mutation bias. To investigate this, a larger set of C_3 angiosperm genomes on Phytozome were analysed to determine whether there was a global, significant, positive association between S_c and GC content and mutation bias.

Three PGLS models were constructed 1) S_c against M_b, and 2) S_c against GC content. In each case, the maximum likelihood value of Pagel's λ estimated from the data was 1. Thus the most likely model assumed an underlying tree that was identical to the species tree and that there was phylogenetic signal in the data. Moreover, as encountered above, as Pagel's λ is equal to 1, the results of the PGLS are identical to those that would be obtained via Felsenstein's phylogenetic independent contrasts[4]. The R^2 between S_c and M_b was 0.56 (p = 2.4 x 10^{-6}). After correcting for phylogeny the R^2 between S_c and M_b decreased to 0.36 (p = 0.0005). Similarly, R^2 between S_c and GC was 0.50 (p = 1.3 x 10^{-5}). After correcting for phylogeny the R^2 between S_c and GC decreased to 0.21 (p = 0.007). Thus, there is a significant association between selection acting on transcript biosynthesis cost and both genome wide GC content and mutation bias. Therefore, the most parsimonious explanation is that variance in PNUE is the major determinant of these traits.

R code for PGLS implementation

```
library("ape")
library("caper")
data<-read.table("data.txt", header=TRUE)
tree<-read.tree("tree.tree")
PGLS <-(comparative.data(phy = tree, data = data, names.col = Species, na.omit =
FALSE, vcv = TRUE, warn.dropped = TRUE))
modelGC.pgls<-pgls(GC ~ Sc, data = PGLS, lambda='ML')
summary(modelGC.pgls)
modelMbCDS.pgls<-pgls(MbCDS ~ Sc, data = PGLS, lambda='ML')
summary(modelMbCDS.pgls)
modelAA.pgls<-pgls(AA ~ Sc, data = PGLS, lambda='ML')
summary(modelAA.pgls)
```

```
modelMbGenome.pgls<-pgls(MbGenome ~ Sc, data = PGLS, lambda='ML')
summary(modelMbGenome.pgls)
```

Data matrix for input to PGLS

Species	GC	MbCDS	MbGenome	Sc	St	AA
Alyrata	0.361	-0.351	-0.572	-0.058	0.013	2.005
Athaliana	0.361	-0.381	-0.573	-0.056	0.010	2.000
Atrichopoda	0.375	-0.280	-0.512	-0.065	0.019	2.017
Bdistachyon	0.464	0.409	-0.144	-0.030	0.008	2.014
Brapa	0.352	-0.201	-0.610	-0.049	0.009	1.991
Cclementina	0.350	-0.439	-0.621	-0.058	0.017	2.002
Cgrandiflora	0.365	-0.306	-0.554	-0.055	0.035	2.001
Cpapaya	0.349	-0.386	-0.622	-0.044	0.023	2.010
Crubella	0.356	-0.340	-0.591	-0.055	0.015	2.001
Csativus	0.324	-0.449	-0.735	-0.069	0.018	1.995
Csinensis	0.348	-0.431	-0.629	-0.052	0.033	2.008
Egrandis	0.393	0.004	-0.435	-0.030	0.011	2.022
Esalsugineum	0.377	-0.268	-0.502	-0.049	0.009	1.999
Fvesca	0.384	-0.262	-0.475	-0.047	0.019	2.012
Graimondii	0.332	-0.450	-0.698	-0.065	0.007	1.987
Lusitatissimum	0.396	-0.071	-0.424	-0.036	0.008	2.004
Mdomestica	0.380	-0.183	-0.490	-0.053	0.008	1.999
Mesculenta	0.359	-0.469	-0.578	-0.064	0.011	1.996
Mguttatus	0.354	-0.169	-0.600	-0.036	0.004	1.991
Osativa	0.436	0.393	-0.259	-0.018	0.006	2.012
Ppersica	0.375	-0.314	-0.510	-0.055	0.017	2.008
Ptrichocarpa	0.337	-0.479	-0.675	-0.045	0.015	2.001
Rcommunis	0.338	-0.466	-0.670	-0.062	0.013	2.003
Slycopersicum	0.341	-0.624	-0.661	-0.049	0.010	2.002
Spolyrhiza	0.420	0.606	-0.321	-0.030	0.014	2.034
Stuberosum	0.348	-0.582	-0.628	-0.049	0.010	1.998
Tcacao	0.340	-0.448	-0.663	-0.048	0.025	2.001
Vvinifera	0.345	-0.335	-0.639	-0.057	0.012	2.011

Phylogenetic tree for input to PGLS

((((((((((((Alyrata:0.01097,Athaliana:0.01298)N39:0.00671,(Crubella:0.00455,Cgrandiflora:0.00221)N40:0.01635)N37:0.01046,(Brapa:0.03458,Esalsugineum:0.01944)N38:0.00694)N36:0.08553,Cpapaya:0.08192)N33:0.00995,(Graimondii:0.04185,Tcacao:0.03071)N34:0.03508)N29:0.00393,(Cclementina:0.00438,Csinensis:0.00699)N30:0.06921)N24:0.00444,Egrandis:0.10073)N19:0.00187,(((((Mdomestica:0.056,Ppersica:0.02797)N35:0.0128,Fvesca:0.07012)N32:0.03156,Csativus:0.10315)N28:0.00509)N23:0.00601,((Mesculenta:0.04332,Rcommunis:0.05426)N25:0.01403,(Ptrichocarpa:0.05947,Lusitatissimum:0.12791)N26:0.00488)N22:0.01716)N18:0.00192)N14:0.00783,Vvinifera:0.06537)N11:0.00718,((Slycopersicum:0.0109,Stuberosum:0.01157)N15:0.07595,Mguttatus:0.10338)N12:0.02727)N9:0.02627,(((Osativa:0.04786,Bdistachyon:0.05704)N17:0.00992)N13:0.11425,Spolyrhiza:0.14335)N10:0.02393)N8:0.02673,Atrichopoda:0.13856)N6;

Comparing K_a, K_s and S_c

It has previously been shown that variation in the strength of selection acting on transcript biosynthesis cost can cause a corresponding variation in the evolutionary rate of gene sequences in bacteria[5]. To determine whether a similar phenomenon occurred in plants a comparative analysis of K_a, $K_{s,}$ and *Sc* was conducted. As described in the methods the predicted proteins from 38 plant species were downloaded from Phytozome[6]. These species were subject to orthogroup and ortholog inference using OrthoFinder[7]. All 1406 pairwise comparisons between species were subsequently conducted. Each pairwise comparison comprised the following steps. 1) The full set of single copy orthologs for the species pair under consideration were isolated. 2) The protein sequences for each orthologous pair were aligned using MAFFT[8] L-INS-i and the coding sequences re-threaded back through the protein sequence alignment. 3) The resulting coding sequence alignments were parsed to remove any gap-containing columns. 4) Un-gapped alignments containing more than 100 aligned codons were subject to K_a and K_s inference using KaKsCalculator v2.0[9] using the default settings. As both K_a and K_s are driven by the same mutational process but subject to different selective forces it is expected K_a and K_s will be linearly related. The anticipated linear relationship was observed for pairwise comparisons where mean K_s was ≤ 1 (Figure S1A). However, for values ≥1 K_s is saturated and estimates of K_s are unreliable (Figure S1A). Thus for analysis of Ks only species comparisons where the mean K_s of all orthologs in the comparison is ≤ 1 were selected for analysis. As K_a did not saturate, no such filtering was applied to the analysis of the K_a data.

The proportion of variance in K_s that could be explained by variation in S_c was analysed as a function of mean K_s between species. This revealed that on average variance in S_c could explain ~10% of variance in K_s between orthologs for species comparisons where mean K_s was ≤ 1 (Figure S1B). However, for species comparisons where mean K_s was ≥ 1, and K_s estimates are unreliable, this explanatory power decreased towards zero as mean K_s increased (Figure S1B). In contrast, variance in S_c explained ~2% of variance in K_a irrespective of mean K_a between species (Figure S1C).

Comparing Mb estimated from gene regions using CodonMuSe with Mb estimated from genome sequence with no current annotation

Mutation bias in transcribed regions of the genome is different from the mutation bias observed in non-transcribed regions of the genome[10]. Accordingly, M_b estimated from coding sequences using CodonMuSe is different from Mb estimated from genome sequences. However, as expected these two estimates correlate very well ($R^2 = 0.85$, $p \leq 10^{-16}$, Figure S2).

The PGLS for the 11 species with PNUE data using M_b estimated from genome sequence obtained an analogous result to the one reported above. There is no significant relationship between MbGenome and PNUE when phylogeny is taken into consideration ($R^2 = 0.10$, $p = 0.23$). To exclude the possibility that low sample size caused the statistical association between either PNUE and genome-wide mutation bias to fail, an additional analysis on a larger species set was conducted exactly as before. Analogous to the result obtained for M_b obtained from CDS sequences, the R^2 between S_c and MbGenome was 0.52 ($p = 1.3 \times 10^{-5}$). After correcting for phylogeny the was still a significant position interaction between S_c and M_bGenome decreased to ($R^2 = 0.22$, $p = 0.0069$). Therefore, as above, the most parsimonious explanation is that variance in PNUE is also a determinant of genome wide mutation bias.

The R code to repeat this analysis and complete datasets for the 11 species and larger datasets are provided above.

Figures

Figure S1

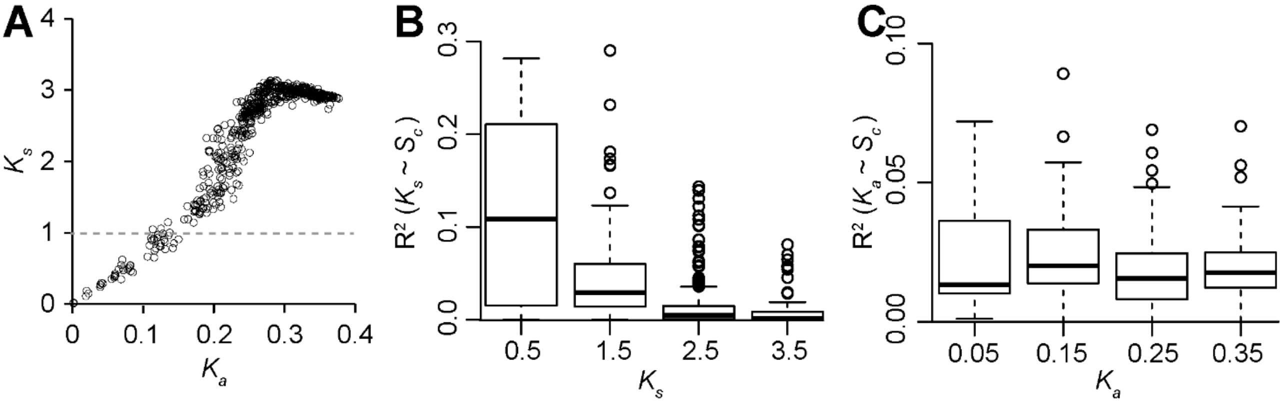

Figure S1. Variance in S_c can explain variance in molecular evolution rate. A) Plot of mean K_a vs mean K_s for all 1406 pairwise species comparisons. B) Plot of proportion of variance in K_s that is explainable by variance in S_c as a function of mean K_s between species. C) Plot of proportion of variance in K_a that is explainable by variance in S_c as a function of mean K_a between species.

Figure S2

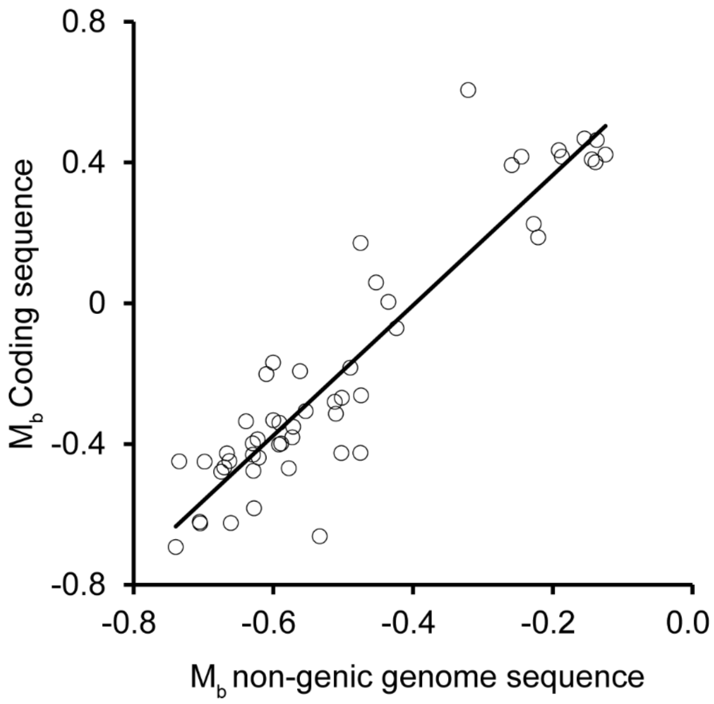

Figure S2. The correlation between Mb estimated from genome sequences with no annotated features with Mb estimated from coding sequences by CodonMuSe. Here values below zero indicate mutation bias towards AT and values above zero indicate mutation bias towards GC.

References

1 Keck, F., Rimet, F., Bouchez, A. & Franc, A. phylosignal: an R package to measure, test, and explore the phylogenetic signal. *Ecology and evolution* **6**, 2774-2780, doi:10.1002/ece3.2051 (2016).

2 Martins, E. P. & Hansen, T. F. Phylogenies and the Comparative Method: A General Approach to Incorporating Phylogenetic Information into the Analysis of Interspecific Data. *The American Naturalist* **149**, 646-667 (1997).

3 Orme, D. caper: Comparative Analysis of Phylogenetics and Evolution in R. (2012).

4 Felsenstein, J. Phylogenies and the Comparative Method. *The American Naturalist* **125**, 1-15 (1985).

5 Seward, E. A. & Kelly, S. Selection-Driven Cost-Efficiency Optimisation Of Transcript Sequences Determines The Rate Of Gene Sequence Evolution In Bacteria. *bioRxiv*, doi:10.1101/136861 (2017).

6 Goodstein, D. M. *et al.* Phytozome: a comparative platform for green plant genomics. *Nucleic acids research* **40**, D1178-1186, doi:10.1093/nar/gkr944 (2012).

7 Emms, D. M. & Kelly, S. OrthoFinder: solving fundamental biases in whole genome comparisons dramatically improves orthogroup inference accuracy. *Genome biology* **16**, 157, doi:10.1186/s13059-015-0721-2 (2015).

8 Katoh, K., Kuma, K., Miyata, T. & Toh, H. Improvement in the accuracy of multiple sequence alignment program MAFFT. *Genome informatics. International Conference on Genome Informatics* **16**, 22-33 (2005).

9 Wang, D., Zhang, Y., Zhang, Z., Zhu, J. & Yu, J. KaKs_Calculator 2.0: a toolkit incorporating gamma-series methods and sliding window strategies. *Genomics, proteomics & bioinformatics* **8**, 77-80, doi:10.1016/S1672-0229(10)60008-3 (2010).

10 Belfield, E. J. *et al.* DNA mismatch repair preferentially protects genes from mutation. *Genome research* **28**, 66-74, doi:10.1101/gr.219303.116 (2018).
