# Supplementary material for: The Amount of Nitrogen Used for Photosynthesis Modulates Molecular Evolution in Plants
Source: Mol Biol Evol. 2018 Apr 19;35(7):1616–25. doi: 10.1093/molbev/msy043 (PMC5995192; doi:10.1093/molbev/msy043)
Supplement: Supplementary Data [file msy043_supp.zip › Supplemental_File_S4.pdf]

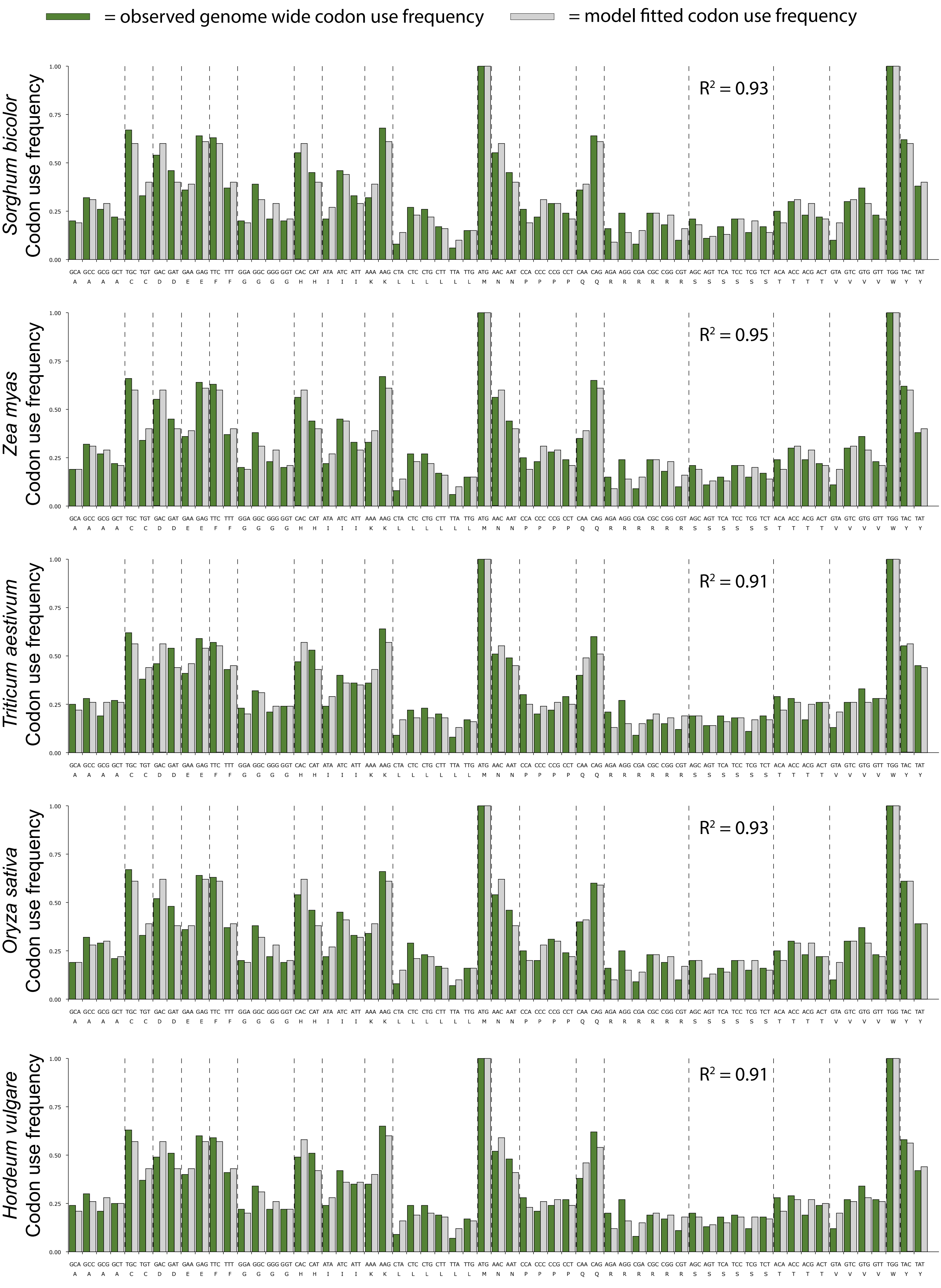
= observed genome wide codon use frequency
= model fitted codon use frequency
Sorghum bicolor
Codon use frequency
$R^2 = 0.93$
Zea myas
Codon use frequency
$R^2 = 0.95$
Triticum aestivum
Codon use frequency
$R^2 = 0.91$
Oryza sativa
Codon use frequency
$R^2 = 0.93$
Hordeum vulgare
Codon use frequency
$R^2 = 0.91$
0.00 0.25 0.50 0.75 1.00
GCA GCC GCG GCT TGC TGT GAC GAT GAA GAG TTC TTT GGA GGC GGG GGT CAC CAT ATA ATC ATT AAA AAG CTA CTC CTG CTT TTA TTG ATG AAC AAT CCA CCC CCG CCT CAA CAG AGA AGG CGA CGC CGG CGT AGC AGT TCA TCC TCG TCT ACA ACC ACG ACT GTA GTC GTG GTT TGG TAC TAT
A A A A C C D D E E F F G G G G H H I I I K K L L L L L L M N N P P P P Q Q R R R R R R S S S S S S T T T T V V V V W Y Y

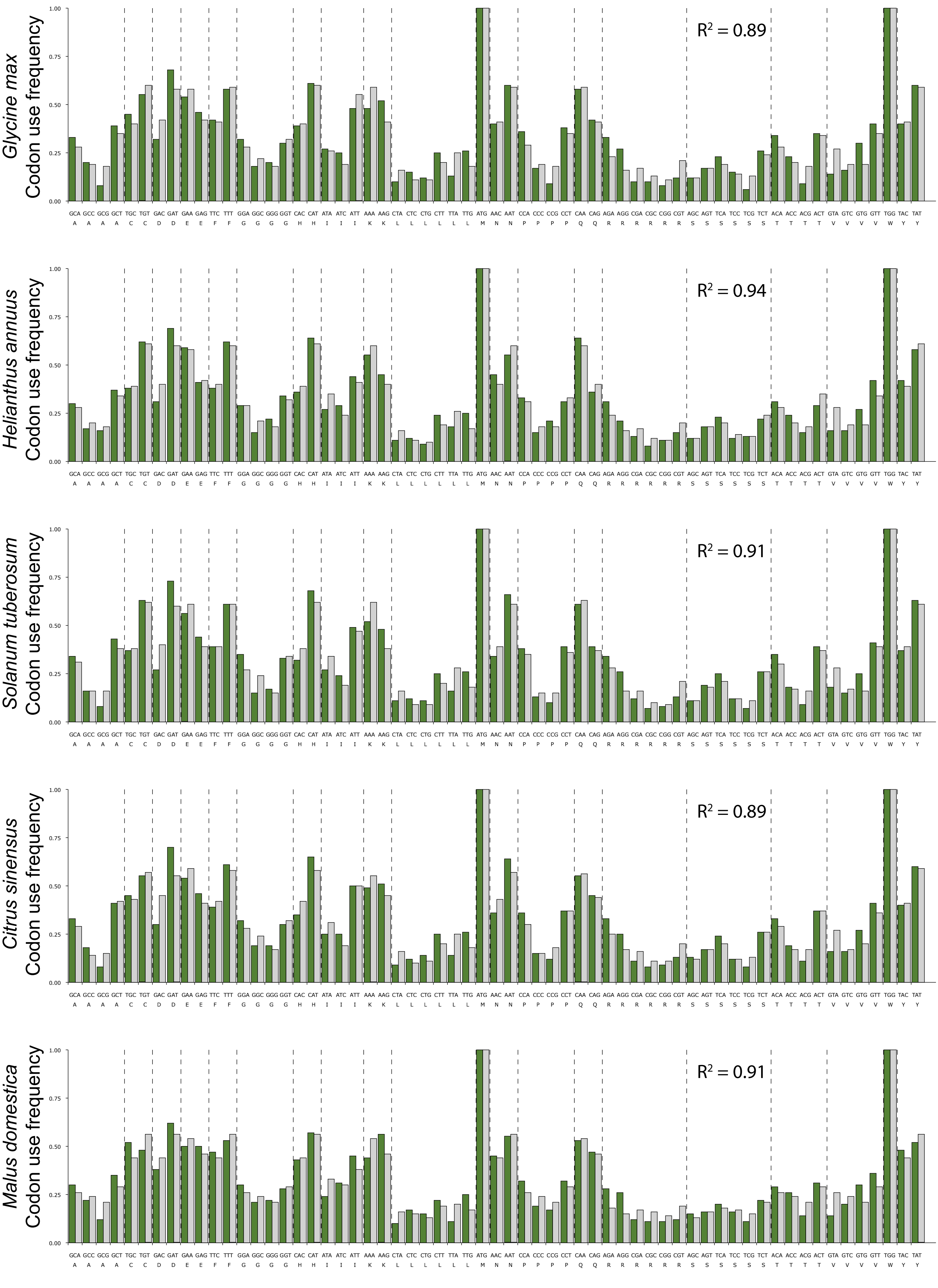
Glycine max
Codon use frequency
R^2 = 0.89
Helianthus annuus
Codon use frequency
R^2 = 0.94
Solanum tuberosum
Codon use frequency
R^2 = 0.91
Citrus sinensus
Codon use frequency
R^2 = 0.89
Malus domestica
Codon use frequency
R^2 = 0.91
0.00 0.25 0.50 0.75 1.00
GCA GCC GCG GCT TGC TGT GAC GAT GAA GAG TTC TTT GGA GGC GGG GGT CAC CAT ATA ATC ATT AAA AAG CTA CTC CTG CTT TTA TTG ATG AAC AAT CCA CCC CCG CCT CAA CAG AGA AGG CGA CGC CGG CGT AGC AGT TCA TCC TCG TCT ACA ACC ACG ACT GTA GTC GTG GTT TGG TAC TAT
A A A A C C D D E E F F G G G G H H I I I K K L L L L L L M N N P P P P Q Q R R R R R R S S S S S S T T T T V V V V W Y Y

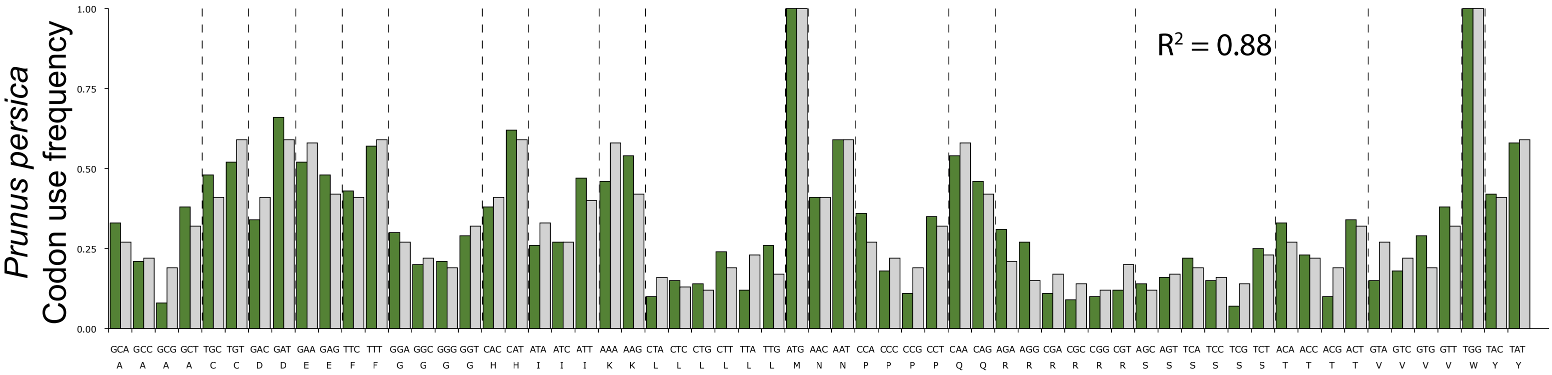

Prunus persica
Codon use frequency
R² = 0.88
1.00
0.75
0.50
0.25
0.00
GCA GCC GCG GCT TGC TGT GAC GAT GAA GAG TTC TTT GGA GGC GGG GGT CAC CAT ATA ATC ATT AAA AAG CTA CTC CTG CTT TTA TTG ATG AAC AAT CCA CCC CCG CCT CAA CAG AGA AGG CGA CGC CGG CGT AGC AGT TCA TCC TCG TCT ACA ACC ACG ACT GTA GTC GTG GTT TGG TAC TAT
A A A A C C D D E E F F G G G G H H I I I K K L L L L L L M N N P P P P Q Q R R R R R R S S S S S S T T T T V V V V W Y Y
