# Supplementary figures and images for: The Amount of Nitrogen Used for Photosynthesis Modulates Molecular Evolution in Plants
Source: Mol Biol Evol. 2018 Apr 19;35(7):1616–25. doi: 10.1093/molbev/msy043 (PMC5995192; doi:10.1093/molbev/msy043)

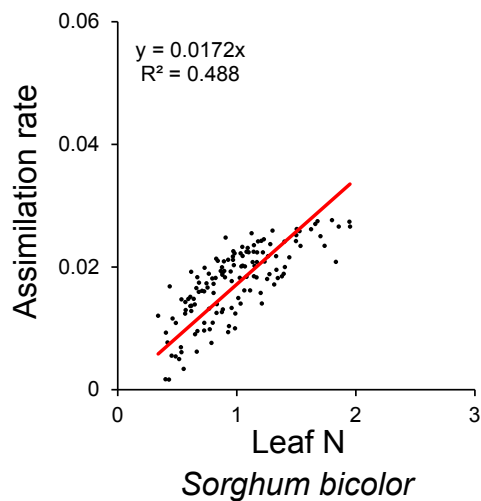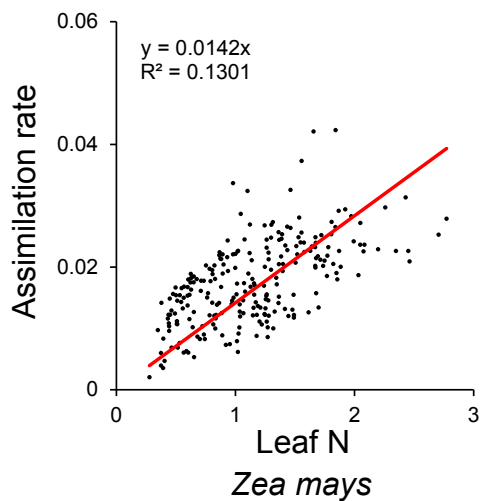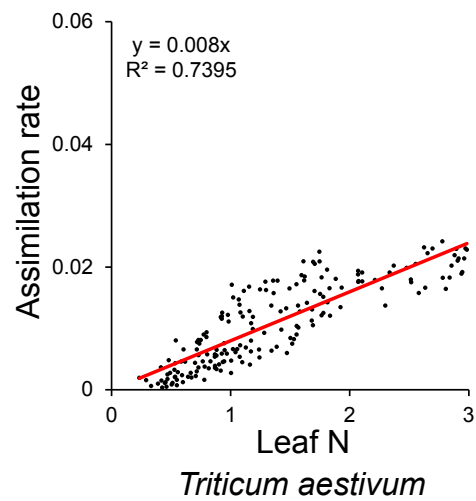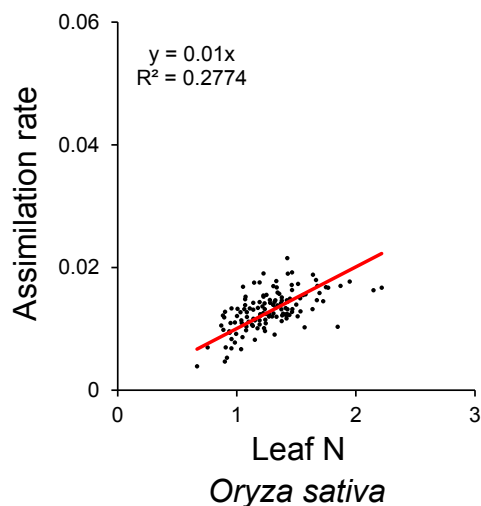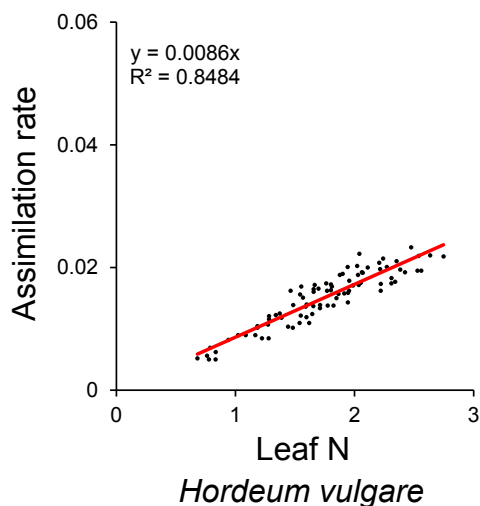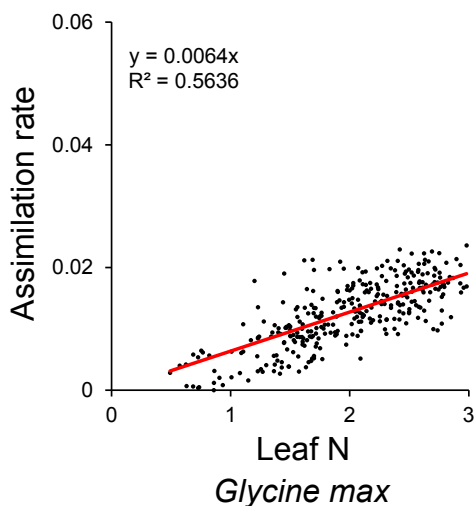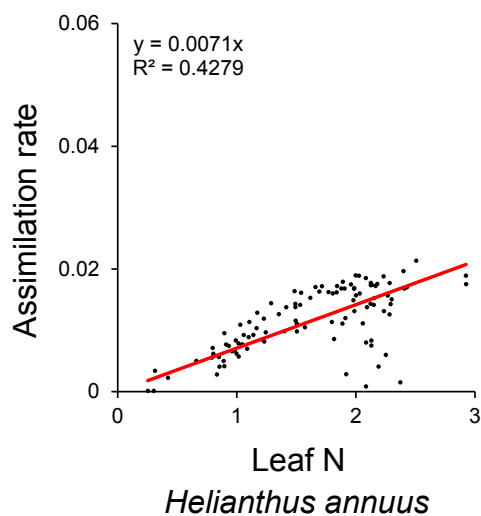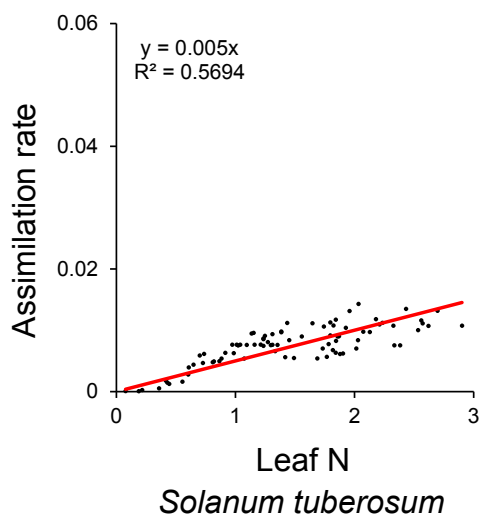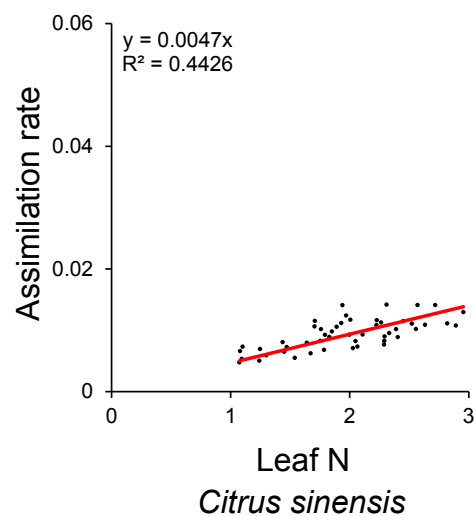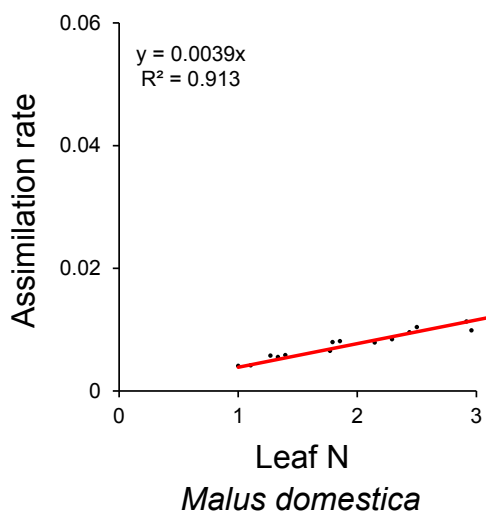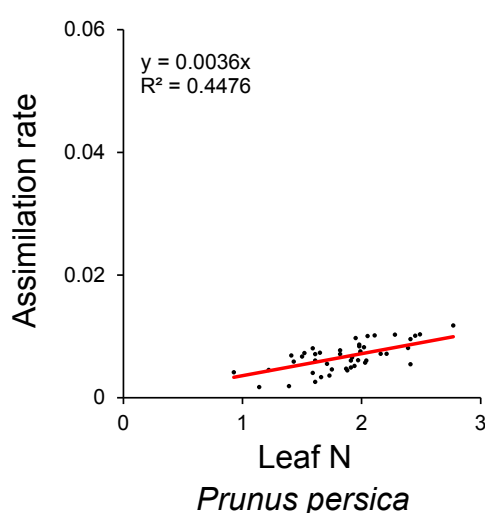

Supplement: Supplementary Data [file msy043_supp.zip › Supplemental_File_S1.pdf]

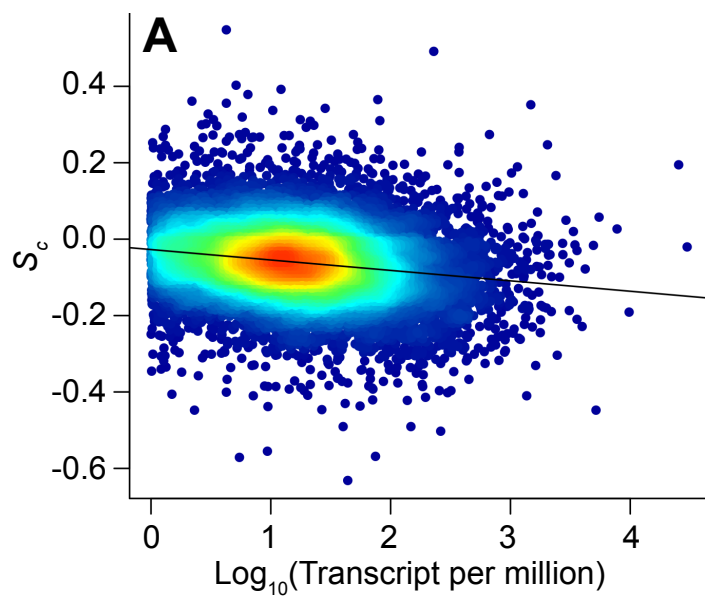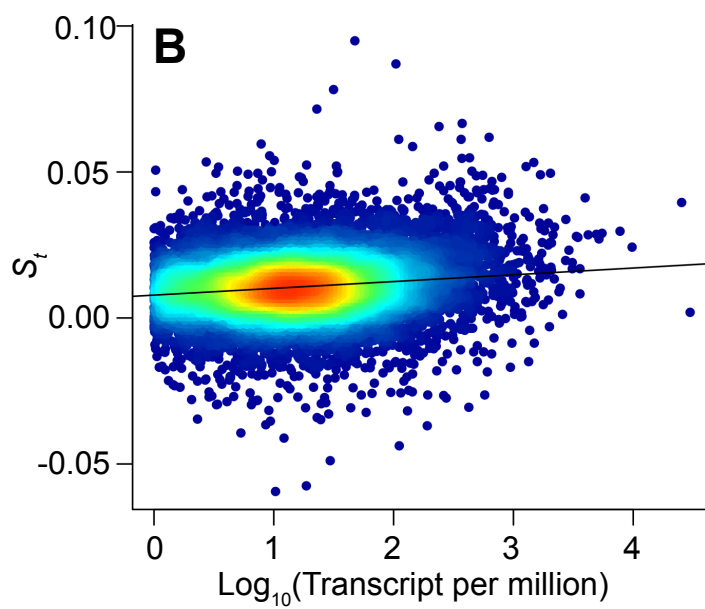

Supplement: Supplementary Data [file msy043_supp.zip › Supplemental_File_S3.pdf]
